# Prevalence of missed canals in endodontically treated teeth: A cone-beam computed tomography study

**DOI:** 10.4317/jced.60282

**Published:** 2023-08-01

**Authors:** Armita Rouhani, Seyed-MohammadReza Aboutorabzadeh, Morteza Reyhani, Nasir Kheirabadi, Samare Mortazavi, Sara Navabi

**Affiliations:** 1 DDS, MSC. Department of Endodontics, Mashhad University of Medical Sciences, Faculty of Dentistry, Mashhad, Iran; 2 DDS. Dentist, Private Practice, Mashhad, Iran; 3 DDS, MSC. Endodontist, Private Practice, Mashhad, Iran; 4 DDS, MSC. Department of Oral and Maxillofacial Radiology, Mashhad University of Medical Sciences, Faculty of Dentistry, Mashhad, Iran; 5 DDS. Dental research center, Mashhad University of Medical Sciences, Mashhad, Iran," and add the new one. Also, the corresponding author's address should be changed to Department of Endodontics, Faculty of Dentistry, Mashhad University of Medical Sciences, Vakilabad Blvd, Mashhad, Iran

## Abstract

**Background:**

This study aimed to measure the prevalence of untreated root canals and periapical lesions in endodontically treated teeth using cone-beam computed tomography (CBCT) and their association with the coronal seal.

**Material and Methods:**

This cross-sectional study assessed CBCT images of studied patients alongside documented information from their dental examinations. The assessment method for analyzing CBCT images after including endodontically treated teeth was the presence of missed canals, and periapical lesions were analyzed in three dimensions.

**Results:**

The evaluations from 772 teeth demonstrated that 89.4% of teeth had the proper coronal seal, 13.3% owned missed root canals, and 29.4% had periapical lesions. Most untreated canals belonged to maxillary first molars (71.8%) and mandibular lateral incisors (33.3%). The prevalence of periapical lesions was highest in mandibular lateral incisors (58.3%) and maxillary second molars (55.8%). In teeth with missed canals, the most periapical lesions were observed in the first premolars of both jaws. In teeth with the lack of proper coronal seal, the periapical lesions were observed in 55.6% of teeth with untreated root canals.

**Conclusions:**

The results revealed a high prevalence of missed canals and periapical lesions in endodontically treated teeth in our study population.

** Key words:**Untreated Root Canals, Periapical Lesions, 3-D Dental Imaging, Dental Treatment Failure.

## Introduction

The ultimate ideal goal of successful root canal therapy is complete disinfection, removal of the inflamed dental pulp tissue, and prevention of re-entry of microorganisms to dental root canals since teeth maintain functional (1,2). The loss in reaching this goal equals the failure of the whole treatment, which can be multi-factorial (3). Some of these factors, which have close relations, can be the complexity of pulp and root canal systems morphology, missed and untreated canals, root obturation poor quality, non-standard coronal seal and restorations, and varied human errors (2,4). These conditions may result in remaining bacterial infections and periapical lesions (5,6).

One pathway alongside clinical examinations for analysis of the successfulness of root canal therapy is using radiographs. However, 2-dimensional, conventional radiographs could not provide the appropriate level of posttreatment analysis. Therefore, cone-beam computed tomography (CBCT) imaging, which is 3-dimensional with high resolution, has been introduced to evaluate the treatment quality of endodontically treated teeth more accurately and their association with apical periodontitis (4,7,8). Despite the low cost and lesser x-ray exposures of periapical radiographs for patients, research has demonstrated that the acceptance of CBCT images among endodontists has elevated since this method could assess root canal shapes and locations, periapical pathologies, and root resorption more accurately (2,9-12).

There has been considerable discussion about the significance of finding all the canals present in the root canal system for the best outcome. Additionally, the risks of missed canals have been discussed regarding the prognosis of endodontic treatments. As a result, there is much evidence that there were untreated canals in failed cases that required endodontic retreatment (7,13-15). Nevertheless, epidemiologic studies in this field have problems controlling differences in clinical practice, and numerous prognostic factors are not understood.

This study aimed to measure the prevalence of untreated root canals and periapical lesions in all endodontically treated teeth using CBCT and their association with the coronal seal of teeth of referred patients to the Oral and Maxillofacial Radiology Department, Mashhad University of Medical Sciences.

## Material and Methods

In this cross-sectional study, all CBCT images of patients referred to the Oral and Maxillofacial Radiology Department, Dental School, Mashhad University of Medical Sciences, alongside documented information from their oral and dental examinations by a dentist, who evaluated patients’ restorations, obtained between April and October 2019, were assessed under the local Ethical Committee Code IR.MUMS.DENTISTRY.REC.1399.096. Also, the studied patients signed an informed consent agreement, and patients’ names were kept confidential to the assessors.

In the clinical examination, the examiner evaluated and noted the observations about the clinical coronal seal of teeth. Fit marginal restoration and absence of any restoration or crown fracture with no finding of any decay evidence were considered a proper coronal seal.

CBCT images of the teeth were collected using a Promax 3D Classic CBCT system (Planmeca, Helsinki, Finland) with an exposure setting of 70 kVp, 8 mA, 12 seconds, 160 µm resolution, and a view field of 5 × 8 × 8 cm3. The exclusion criteria for this study were third molars, remained root parts of the teeth, open apex teeth, hopeless teeth with severe root resorption, and deciduous teeth. Likewise, distorted endodontically treated teeth images, which could not be evaluated because of different kinds of artifacts, were excluded. Then, the remaining subjects were analyzed using Romexis Viewer software (Version 4.4.3) by two endodontists, and in disagreement situations, the opinion of an oral and maxillofacial radiologist was considered.

The assessment method for analyzing CBCT images initially included endodontically treated teeth, which means a radiopaque material was seen in at least one root canal beyond the cementoenamel junction (CEJ) (7). Subsequently, the existence of potentially missed canals, root canals without any radiopaque substance from CEJ to the apex or split canals at the coronal, middle, or apical part of the main canal (7), and periapical radiolucencies, when the lamina dura is disrupted, and the area of low density at the apex of the radiograph is more than twice the width of the periodontal ligament space, were analyzed in the axial sections. For the approval of the observations, coronal and sagittal dimensions were subsequently studied. In addition, other collected data for analysis contained sex, age, and number of teeth examined.

Statistical data analysis was performed by IBM SPSS Statistics 16.0 IF006 software. Moreover, the Chi-square and Fisher’s exact tests plus the measurements of the Odds Ratio were run, and statistical significance was determined by *p-value*s less than 0.05.

## Results

In this study, after excluding 4 images with artifacts from 302 CBCT, 298 patients comprised 162 females (54.4%) and 136 males (45.6%) with an average age range of 45.46 ± 12.51 between 15 to 82 years were included, and 772 teeth were assessed (443 (57.4%) maxillary teeth and 329 (42.6%) mandibular teeth). The clinical evaluations demonstrated that 690 (89.4%) teeth had proper coronal seals. Also, observations on CBCT assessment showed that 103 cases (13.3%) owned missed root canals, and in 227 teeth (29.4%), periapical lesions were observed.

**Figure 1:**
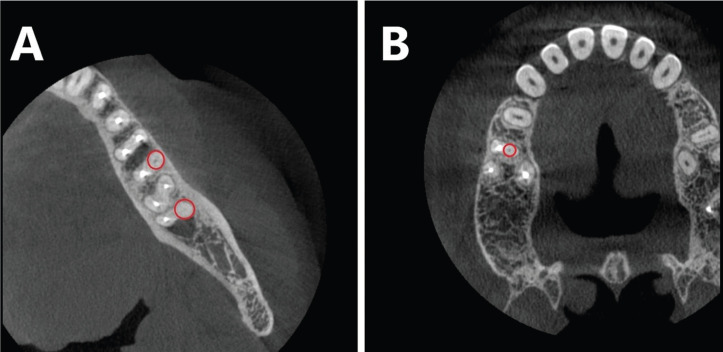
Samples of missed root canals: (A) Missed distobuccal root canals of the first and second molars of the mandible, and (B) The missed second mesiobuccal root canal of the first molar of the maxilla.

The findings showed that in the maxilla, most of the untreated canals belonged to the second mesiobuccal (MB2) canal in the first molars (71.8%). Also, in the mandible, most missed canals were related to lateral incisors (33.3%). In both jaws, the frequency distribution of untreated canals between different teeth was statistically significant (*P* < 0.001) (Table 1, Fig. 1).

**Table 1:**
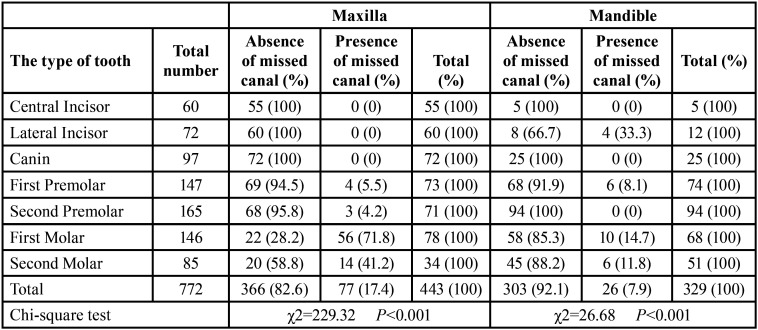
Frequency of missed canals based on tooth type and the jaw.

In the CBCT images, periapical lesions were discovered in 63.5% of teeth with missed canals (66 cases), and 24.1% of completely treated teeth (161 cases), and the difference was statistically significant (*P* < 0.001). Additionally, the observations demonstrated that the prevalence of periapical lesions was highest in mandibular lateral incisors at 58.3% (Table 2).

**Table 2:**
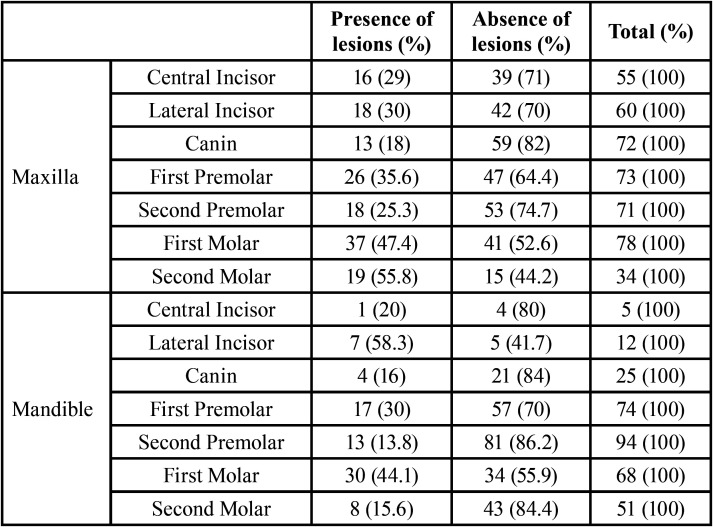
Frequency of periapical lesions in every type of tooth of jaws.

In assessing the relation between missed canals and periapical pathologies, the findings revealed that in the completely treated root canals of teeth, the highest rate of lesions was found in mandibular lateral incisors (50%). Moreover, in teeth with missed canals, most periapical lesions were observed in the first premolars of both jaws (maxilla: 100%, mandible: 83.3%) (Table 3).

**Table 3:**
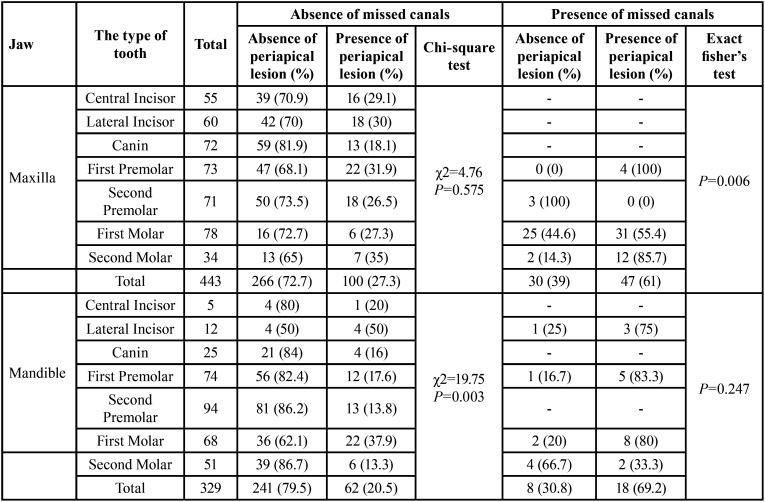
Frequency of periapical lesions based on the presence of missed canals.

The findings of the coronal seal exhibited that in teeth with the lack of proper coronal seal, the difference between the presence or absence of missed canals and the observation of periapical lesions was not statistically significant (*P* = 0.730). On the other hand, in teeth with the proper coronal seal, this difference was statistically significant (*P* < 0.001) (Table 4).

**Table 4:**
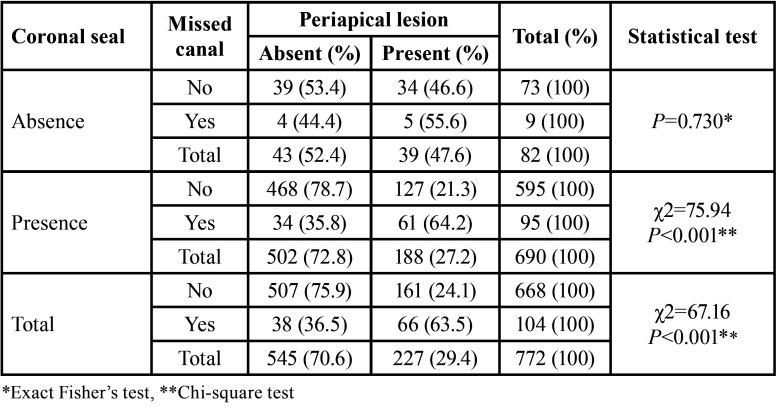
Presence/absence of periapical lesions in the presence/absence of missed canals and coronal seal.

In evaluating the simultaneous effect of variables on the periapical lesion, analyses demonstrated that the chance of remaining or developing these lesions in the teeth without the proper coronal seal was 2.71 times significantly higher than in teeth with the proper coronal seal (*P* = 0.001). Also, the significant chance of remaining or developing periapical pathologies in all root canal-treated teeth was 24% rather than in teeth with untreated root canals (*P* = 0.001). Although this chance in the maxilla was 1.37 times more than in the mandible, it was not significant (*P* = 0.089).

## Discussion

The signs and symptoms of an unsuccessful root canal therapy treatment may vary from the lack of any specific signs to severe apical periodontitis. Untreated root canals in endodontically treated teeth are one of the critical factors of treatment failure. Identifying and finding extra and missed root canals can be challenging due to the complexity of the dental pulp cavity and root canals and the absence of apparent clinical evidence for diagnosing untreated canals (16). Accordingly, this study’s objective was to determine the prevalence of untreated root canals and periapical lesions in endodontically treated teeth using CBCT and assess the association between these lesions and the coronal seal.

The accuracy of CBCT imaging was reported at 98%, which is highly appropriate for evaluating teeth root canal anatomy (17). Also, according to various studies, this method comparing other imaging techniques, was more accurate in finding extra and accessory root canals, periapical pathologies, and alterations in bone tissue (18,19). However, CBCT images are not routinely recommended owing to their higher X-ray dose, cost, unavailability in many areas, and more complicated procedures than conventional intraoral imaging techniques in dental offices. In addition, the presence of some restoration materials and intracanal metal posts could affect the accuracy of CBCT images. As a result, clinicians should utilize other methods and instruments for better observing and finding root canal orifices using proper access cavities, magnifier devices, good lighting, and ultrasonic instruments (20,21).

In this study, the prevalence of missed canals was 13.3%, approximately similar to Costa *et al*. (5) and Baruwa *et al*. (4) studies, in which the prevalence of missed canals was reported at 12.0% and 12.2%, respectively. Other reports for the prevalence of untreated canals differed from this study, such as the 18% prevalence by Mashyakhy *et al*. (22) and 23.04% by Karabucak *et al*. (7). These differences could relate to the assessment of only posterior teeth in their studies.

Moreover, similar to this study that exhibited the most common missed canals in CBCT images found in the MB2 canals of maxillary first molars, other authors like Karabucak *et al*. (7), Costa *et al*. (5), Baruwa *et al*. (4), Mashyakhy *et al*. (22), and do Carmo *et al*. (8) with different prevalence indicated the same results. Considering this, it was recommended that using an endodontic microscope as a magnifier appliance assist the root canal finding process (23,24). In the Buhrley *et al*. study, the finding of the MB2 canal without endodontic microscopes was 18%, whereas observing this canal in the maxillary first molars using the microscope was 57% (25).

In addition, in the current study, the most common untreated canals in the mandible were observed in lateral incisors with a 33.3% prevalence. This commonness could associate with anatomical complexities and the difficulty of preparing a proper access cavity in these teeth (26).

Furthermore, this study presented that the total incidence of periapical lesions was 29.4%, and this incidence for endodontically treated teeth with missed canals was 63.5%. In this regard, different authors’ findings of periapical lesions with CBCT images showed a wide variety of outcomes. For instance, in the Nascimento *et al*. study, the total incidence of periapical lesions was 59.3% (2). Costa *et al*. demonstrated a 98% incidence of these lesions in teeth with untreated canals (5). For the Karabucak *et al*. study, this incidence was 83%, and they reported a 59.5% total incidence of periapical lesions (7). Also, the incidence of periapical pathologies simultaneously with missed canals was 82.6% in the Baruwa *et al*. (4) and 90% in Mashyakhy *et al*. (22) studies. Even though these various incidences could have varied reasons, such as inclusion criteria and assessed teeth in different studies, the significant issue is the high incidence of periapical lesions when a root canal remains untreated. In the Song *et al*. study, the second cause of the observation of these lesions was the existence of missed canals in teeth that had undergone root canal therapies. Untreated canals can be the first location of infections or a suiTable site for secondary infections causing endodontic treatment failure (27).

Besides, analyses of this study on evaluating the simultaneous effect of variables on the periapical lesions demonstrated that the chance of remaining or developing these lesions in the teeth without the proper coronal seal and with untreated canals was significantly higher. Similarly, several studies presented that one of the main reasons for the failure of root canal therapies and changes in periapical conditions was the lack of proper coronal seals (2,28-30).

This study had some limitations, such as not evaluating kappa analysis for calibrating CBCT examiners and not discovering and evaluating other failure factors of endodontic treatments like the quality of obturation and dental treatments’ methods and procedures, whether endodontic-related or post-endodontic in the restoration phase. Also, because of the essence of cross-sectional studies, the possibility for a more accurate assessment of periapical lesions was not accessible since it could be possible that the lesions might be in the healing and size-decreasing phase. Likewise, despite the high accuracy of CBCT imaging, there is a probability of overdiagnosing some periapical lesions. Therefore, it is recommended that future studies perform with more sample size, the documentation of the cause of referring to taking CBCT images, the evaluation of the quality of obturation, and the assessment of treatment methods and materials in the form of a prospective cohort study.

## Conclusions

It can be concluded that the prevalence of missed canals and periapical lesions in endodontically treated teeth was high. Also, the most incidence of missed canals and periapical radiolucencies occurred in the maxillary first molars due to MB2 canals. Furthermore, there was a significant relation between missed canals and coronal seals with periapical lesions. Consequently, according to the relatively high prevalence of untreated canals, especially in the maxillary first molars, identifying failure factors of endodontic treatments and using appropriate techniques, devices, and instruments can decrease errors.
